# Supplementary material for: Serjanic Acid Improves Immunometabolic Markers in a Diet-Induced Obesity Mouse Model
Source: Molecules. 2020 Mar 25;25(7):1486. doi: 10.3390/molecules25071486 (PMC7181135; doi:10.3390/molecules25071486)
Supplement: Supplementary file 1 [file molecules-25-01486-s001.pdf]

***Serjanic acid improve immunometabolic markers in a diet-induced obesity mouse model.***

**SUPPLEMENTARY INFORMATION**

**Table S1. Primers sequence used for RT-PCR expression assays**

| Gene | | Sequences | M.P |
|---|---|---|---|
| PPAR-α | F | 5′- TGTCGAATATGTGGGGACAA-3′ | 59°C |
| | R | 5′- AATCTTGCAGCTCCGATCAC-3′ | |
| ACC | F | 5′- GCCTCTTCCTGACAAACGAG-3′ | 59°C |
| | R | 5′- TGACTGCCGAAACATCTCTG-3′ | |
| PGC-1 | F | 5′- TATGGAGTGACATAGAGTGTGCT-3′ | 59°C |
| | R | 5′- CCACTTCAATCCACCCAGAAAG-3′ | |
| Cpt1a | F | 5′- AGATCAATCGGACCCTAGACAC-3′ | 60°C |
| | R | 5′- CAGCGAGTAGCGCATAGTCA-3′ | |
| Cyclophilin | F | 5′-GGAGATGGCACAGGAGGAA -3′ | 60°C |
| | R | 5′-GTAGTGCTTCAGCTTGAAGTTCTCAT -3′ | |
| TNF-α | F | 5′-CCCACACCGTCAGCCGATTT-3′ | 62°C |
| | R | 5′-GTCTAAGTACTTGGGCAGATTGACC-3′ | |
| IL-6 | F | 5′-CCGGAGAGGAGACTTCACAG-3′ | 62°C |
| | R | 5′-GGAAATTGGGGTAGGAAGGA-3′ | |
| MCP1 | F | 5′-TCAGCCAGATGCAGTTAACGC-3′ | 62°C |
| | R | 5′-TGATCCTCTTGTAGCTCTCCAGC-3′ | |
| IL-1 β | F | 5′-AGAGCTTCAGGCAGGCAGTAT-3′ | 60°C |
| | R | 5′-GAAGGTGCTCATGTCCTCATC-3′ | |

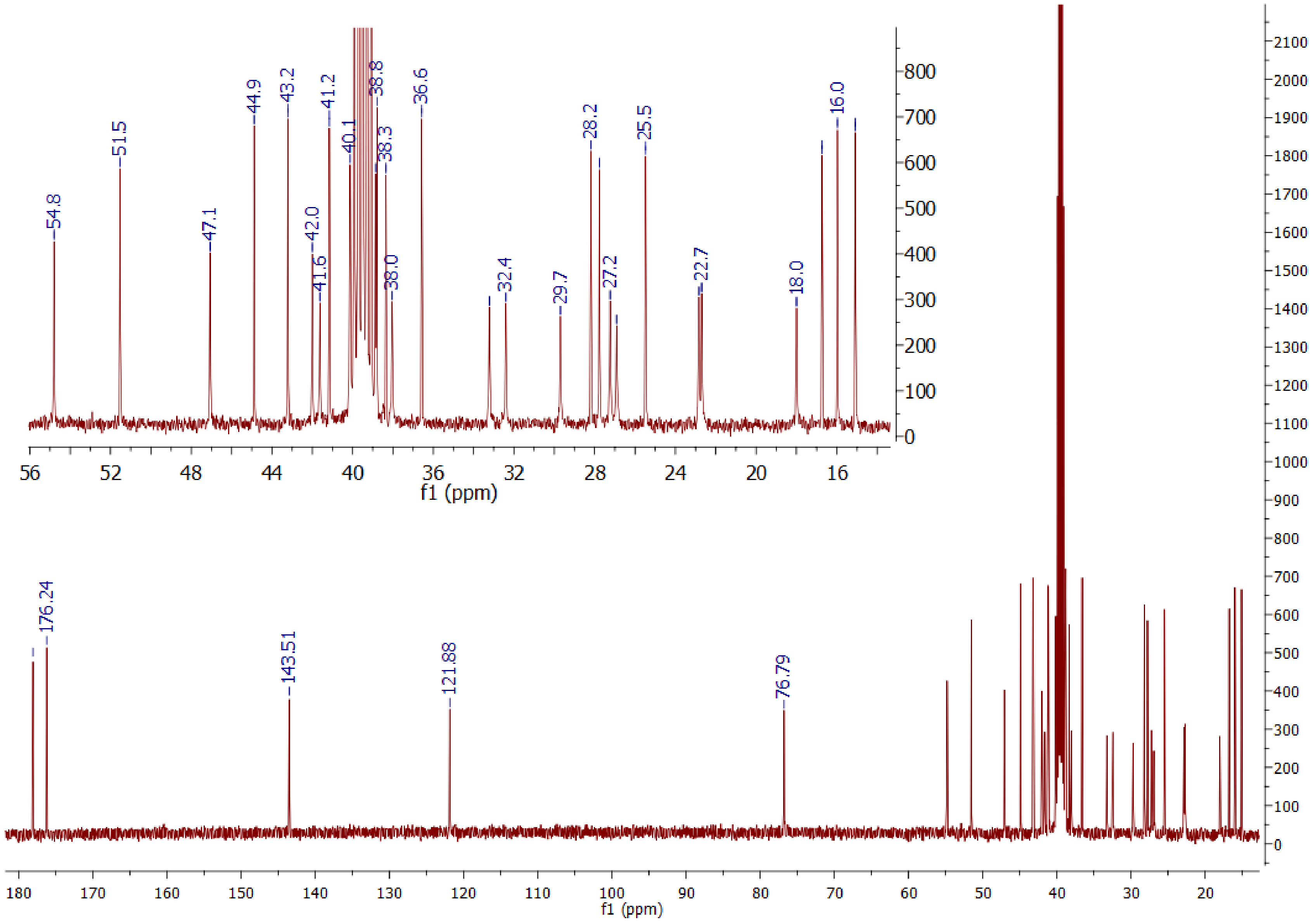


**Figure S1. Serjanic Acid $^{13}$C-NMR spectrum**

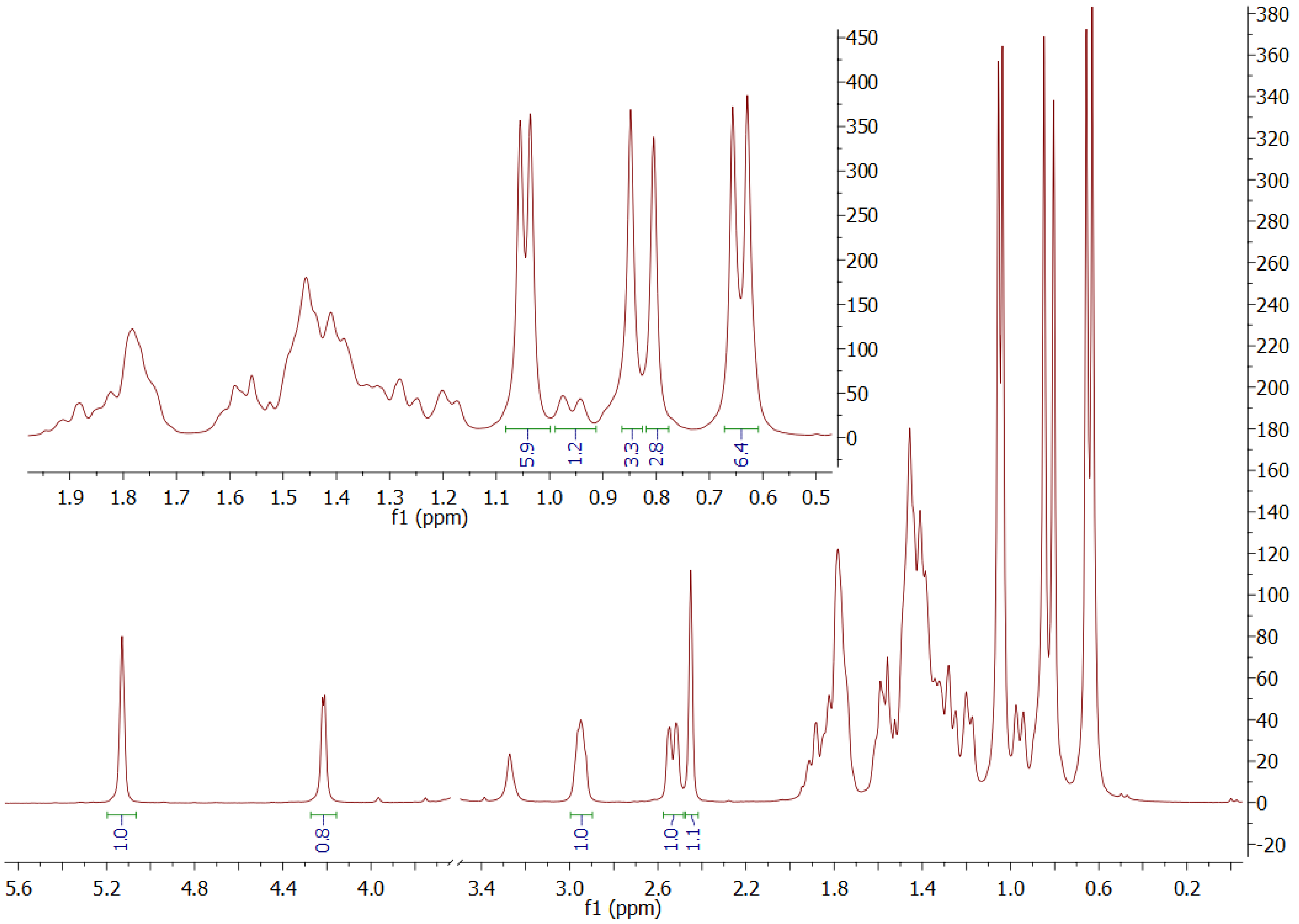


**Figure S2. Serjanic Acid $^{1}$H-NMR spectrum**
